# Correlations between clinical parameters in implant maintenance patients: analysis among healthy and history-of-periodontitis groups

**DOI:** 10.1186/s40729-017-0108-0

**Published:** 2017-10-30

**Authors:** Keisuke Seki, Shinya Nakabayashi, Naoki Tanabe, Atsushi Kamimoto, Yoshiyuki Hagiwara

**Affiliations:** 1 Implant Dentistry, Nihon University School of Dentistry Dental Hospital, Tokyo, 101-8310 Japan; 2 Department of Comprehensive Dentistry and Clinical Education, Nihon University School of Dentistry, 1-8-13 Kanda-Surugadai, Chiyoda-ku, Tokyo, 101-8310 Japan; 3 Department of Applied Mathematics and Informatics, Nihon University School of Dentistry, Tokyo, 101-8310 Japan

**Keywords:** Dental implant, Maintenance, Modified plaque index, Probing depth, Bleeding on probing, Correlation

## Abstract

**Background:**

The pathophysiology and pathology of peri-implantitis remain unclear; however, its similarity to periodontitis has been described. The evaluation of peri-implant tissue and the diagnostic criteria of peri-implant disease are not currently standardized as they are for periodontitis. In this study, we evaluated clinical parameters during the implant maintenance period to determine significant correlations between these parameters.

**Methods:**

We examined 55 implant patients at the time of maintenance visits between April and September 2016 and classified patients into a healthy group (H) and a history-of-periodontitis group (HP). For each implant, we evaluated the modified plaque index, probing pocket depth, and bleeding on probing as clinical parameters. Statistical analyses were performed with Spearman’s rank correlation coefficient.

**Results:**

A total of 130 implants were assessed. The mean time since implant placement was 6 years and 6 months. The prevalence of implant-based peri-implantitis was 10.8% of all the implants. All cases of implant-based peri-implantitis came from the HP group, and many were present in patients with a history of severe periodontitis. The probing pocket depth around the implant was significantly greater in the HP group than in the H group. We found weak positive correlations between the probing pocket depth and bleeding on probing (*r*_*s*_ = 0.401, *p* < 0.05) in the H group and between the probing pocket depth and bleeding on probing (*r*_*s*_ = 0.241, *p* < 0.05) and the modified plaque index (*r*_*s*_ = 0.228, *p* < 0.05) in the HP group.

**Conclusions:**

Our findings suggest that probing pocket depth and bleeding on probing as clinical parameters are important indicators for the diagnosis of peri-implant disease during the maintenance period among healthy and history-of-periodontitis groups.

## Background

Peri-implant diseases are the main biological complication of implant treatment and greatly influence treatment success [1]. Risk indicators for peri-implantitis include a history of periodontitis, poor oral hygiene, and smoking [2–4]. Peri-implantitis is often encountered clinically, occurring in 12 to 43% of implants and 28 to 47% of implant patients [5–8]. Similarities between the pathology and pathogenic factors of peri-implantitis and those of periodontal disease (such as periodontitis) have been described [9–11]; however, many points remain unclear [12]. For this reason, discussion is ongoing about the clinical criteria and selection of treatment options for peri-implantitis [13, 14].

The diagnostic criteria and treatments for peri-implant diseases such as peri-implantitis are not currently standardized. In contrast, periodontitis is diagnosed by periodontal tissue probing, attachment level, the status of oral hygiene, tooth mobility, occlusion, and furcation and dental X-ray imaging.

Ideally, peri-implant mucositis is discovered in its early stages to allow longer maintenance therapy, preventing complications and maintaining a high success rate [15]. No previous study describing clinical examination, such as probing the tissue surrounding implants, has been reported in Japanese patients. In this study, we statistically evaluated various clinical examination parameters during implant maintenance to determine correlations between them.

## Methods

### Selection of patients

This study enrolled 55 patients (25 men, 30 women; mean age, 63.53 ± 10.51 years) who visited Nihon University School of Dentistry Dental Hospital for implant maintenance from April to September 2016. The inclusion criteria were treatment at the same hospital with two-stage implant placement before 2015, and ongoing maintenance at 3–6-month intervals after superstructure placement. Patients whose superstructure had been in place for less than 3 months were excluded. The dental implants used were Replace Select™/Steri-Oss® implant system (Novelbiocare, Switzerland), Novel Replace® (Novelbiocare, Switzerland), OsseoSpeed™ (ASTRA TECH Implant System, Switzerland), OSSEOTITE® XP (BIOMET 3i, Indiana, USA), and Brånemark system® Mk III (Novelbiocare, Switzerland); all implants had a rough surface (Table 1). This study was conducted with the approval of the Nihon University School of Dentistry Ethics Committee (Permit Number: 2013-15).

**Table 1:** Implant systems used

Implant	Number
Replace select ™	44
Novel Replace ®	31
OsseoSpeed ™	25
OSSEOTITE ® XP	18
Brånemark system® Mk III	7
Steri-Oss ® implant system	5
Total	130

### History of periodontitis before implant treatment

Patients with chronic periodontitis were diagnosed based on the latest AAP Classification of Periodontal Diseases and Conditions at the first visit. Clinical attachment level (CAL) was examined for each individual tooth. A CAL of 1–2 mm was defined as mild periodontitis, 3–4 mm was moderate periodontitis, and 5 mm or more was severe periodontitis. The range of the disease was considered to be localized when 30% or less of all teeth were affected, and generalized when more than 30% of teeth were affected [16]. Cases of aggressive periodontitis were not included in this study. Implant therapy was performed after active periodontal treatment with confirmed improvement of the periodontal tissue. We classified patients into two groups based on their medical records: a healthy group without a history-of-periodontitis (H) and a history-of-periodontitis group (HP).

### Dental history

We recorded the following information from the medical chart: patient sex and age, number of natural teeth at first visit, final number of natural teeth at the beginning of implant treatment, total number of extracted teeth and installed implants, and average maintenance period.

### Diagnosis of peri-implantitis

At the time of maintenance, the status of the peri-implant tissue was evaluated. Peri-implantitis is defined as a chronic infection of peri-implant tissue, with resorption of the supporting bone observable radiographically. Peri-implant mucositis is a reversible inflammatory reaction around the implant [17]. We diagnosed peri-implantitis when the probing pocket depth (PPD) around the implant was 6 mm or more, suppuration and bleeding were observed at the time of probing, and bone resorption was present radiographically for 25% or more of the implant length [18].

### Maintenance examinations

At the time of optional maintenance, the following parameters were measured for each implant. Data collection and analysis were conducted by two operators who were blind to the research purpose and methods. One operator statistically analyzed the final data set.

#### Evaluation of plaque adhesion

Plaque adhesion (modified Plaque Index: mPI) on the superstructure of the implant was measured on a 4-point scale (0, no plaque; 1, plaque seen with probing of the tooth surface; 2, moderate accumulation of soft deposits visible with the naked eye; 3, abundance of soft deposits on the tooth). We calculated the average score of four surfaces (buccal, lingual, mesial, and distal aspects).

#### Probing pocket depth around implants

To standardize the probing force (0.15 N), the operators used a precision scale to calibrate their force with repeated measurements. The same operator inserted a periodontal pocket probe (11 Colorvue® Probe Kit; Hu-Friedy, Chicago, IL, USA) into the pocket around the implant with a force of approximately 0.15 N. PPD was measured in 1-mm increments with the 6-point method (at the mesial and distal angles and at the center of the buccal and lingual aspects of the implant). We calculated the average of the six points for each implant.

#### Bleeding on probing of peri-implant mucosa

Bleeding on probing (BoP) was evaluated 10 s after the 6-point probing as negative (0, no bleeding) or positive (1, bleeding). We calculated the average of all six points obtained. The minimum BoP score was 0 and the maximum was 1.

### Statistical analysis

Data analysis was performed with statistical software (GraphPad Prism 5.0; GraphPad Software Inc., San Diego, CA, USA). Quantitative analysis of differences between the H and HP groups was performed with the Mann–Whitney *U* test. Clinical parameters were analyzed with Spearman’s rank correlation coefficient (*R*_*s*_). A significance level of 95% (*p* < 0.05) was considered statistically significant.

## Results

### Patients

Of the 55 subjects, 25 were male and 30 were female (H group: 8/10, HP group: 17/20, respectively). A total of 130 implants (H group 39, HP group 91) were included in the analysis (Table 2). The mean maintenance period was 6 years and 6 months. The average number of natural teeth at the first visit and at the beginning of implant treatment were 25.3 ± 2.9 and 22.4 ± 4.5, respectively. Comparison between the H and HP groups revealed a significant difference in patient age at the time of maintenance, number of natural teeth at the beginning of implant treatment, total number of extracted teeth, and time since implant placement.

**Table 2:** Characteristics of the study population

	H group mean ± SD, or *n* (median)	HP group mean ± SD, or *n* (median)	Significant difference between H and HP group
**Age**	56.0 ± 11.8 (57.9)	67.2 ± 7.7 (66.9)	**
**Number of teeth** (at the first visit) (at the beginning of implant treatment)	25.3 ± 3.0 (26.5)	25.2 ± 2.9 (26.0)	n.s.
	23.9 ± 3.6 (25.0)	21.6 ± 4.7 (23.0)	*
**Number of extracted teeth**	1.6 ± 1.2 (1.0)	4.1 ± 4.0 (2.0)	**
**Maintenance period**	4 years 3 months (5 years 8 months)	7 years 4 months (8 years 4 months)	**

*n.s.* not significant, *p < 0.05, **p < 0.01

### Prevalence of peri-implantitis, history of periodontitis severity

Among all the subjects, 14 implants (10.8%) had peri-implantitis, all belonging to the HP group (6 patients). Details of periodontitis diagnosis in the HP group were generalized moderate periodontitis (22 patients, 53 implants), generalized moderate periodontitis + localized severe periodontitis (11 patients, 23 implants), and generalized severe periodontitis (4 patients, 15 implants) (Table 3). The proportion of peri-implantitis for each diagnosis were 5.7% (3/53) for generalized moderate periodontitis, 13.0% (3/23) for generalized moderate periodontitis + localized severe periodontitis, and 53.3% (8/15) for generalized severe periodontitis at the implant level. The prevalence of peri-implantitis in the HP group was 15.4% (14/91 at the implant level) and 16.2% (3/37 at the patient level).

**Table 3:** Prevalence of peri-implantitis and periodontitis diagnosis in the HP group

Diagnosis in HP group (37 patients, 91 implants) Localized: ≤ 30% of site involved, generalized: > 30% of site involved		Numbers of peri-implantitis and prevalence in diagnosis *n* = 14, 15.4% of HP group
Diagnosis of chronic periodontitis	Implants	Peri-implantitis implants
Generalized moderate (22 patients)	53	3 (5.7 %)
Generalized moderate + Localized severe (11 patients)	23	3 (13.0 %)
Generalized severe (4 patients)	15	8 (53.3%)

### Clinical parameters

The data from each subgroup are shown in Table 4.

**Table 4:** Value of each clinical parameter

Clinical parameters	H group (*n* = 39) mean ± SD/median	HP group (*n* = 91) mean ± SD/median	Significance difference between H and HP group
mPI	0.18 ± 0.23/0.25	0.23 ± 0.33/0.25	n.s.
PPD	2.87 ± 0.48/2.83	3.33 ± 1.07/3.00	**
BoP	0.30 ± 0.28/0.17	0.28 ± 0.29/0.17	n.s.

*n.s.* not significant, *p < 0.05, **p < 0.01

### Modified plaque index

The mPI score for the total study population was 0.21 ± 0.30 (mean ± SD). The mean scores of the H and HP groups were 0.18 (median, 0.25) and 0.23 (median, 0.25), respectively.

### Probing pocket depth

The PPD of the total study population was 3.19 ± 0.96 mm (mean ± SD). The mean PPDs of the H and HP groups were 2.87 mm (median, 2.83 mm) and 3.33 mm (median, 3.00 mm), respectively.

### Bleeding on probing

The BoP score of the total study population was 0.28 ± 0.29 (mean ± SD). The mean scores of the H and HP groups were 0.30 (median, 0.17) and 0.28 (median, 0.17), respectively.

### Correlations between PPD and characteristics of the study population

Because normality and homoscedasticity were not obtained for each clinical parameter between the two groups, a statistical study was conducted using the nonparametric test (Mann–Whitney *U* test) for the difference test between the two groups. PPD was recognized to be significantly greater (*p* < 0.01) in the HP group, but there were no significant differences in mPI and BoP between the two groups. Therefore, we focused on the PPD parameter, in which a significant difference was found between the two groups, and analyzed correlations between PPD and patient characteristics (age, number of teeth, number of extracted teeth, maintenance period). Significant correlation coefficients were observed for the number of extracted teeth in the H group and for all characteristics in the HP group (Table 5).

**Table 5:** Correlations between PPD and characteristics

Characteristics	H group correlation (*R*_*s*_)	HP group correlation (*R*_*s*_)
**Age**	0.040 n.s.	0.209^*^
**Number of teeth** (at the beginning of implant treatment)	0.027 n.s.	− 0.355^**^
**Number of extracted teeth**	0.369^*^	0.284^**^
**Maintenance period (month)**	0.193 n.s.	0.234*

*n.s.* not significant, * *p* < 0.05, **p < 0.01

### Comparison of clinical parameters between the H and HP groups

We analyzed the correlation between PPD and other parameters in each group using Spearman’s rank correlation coefficient. We found a weak correlation between PPD and BoP for the H group (*r*_*s*_ = 0.401, *p* < 0.05). In the HP group, weak correlations were found between PPD and mPI (*r*_*s*_ = 0.228, *p* < 0.05) and between PPD and BoP (*r*_*s*_ = 0.241, *p* < 0.05) (Table 6).

**Table 6:** Correlations between PPD and the other parameters

Parameters	H group correlation (*R*_*s*_)	HP group correlation (*R*_*s*_)
PPD-mPI	0.182 n.s.	0.228^*^
PPD-BoP	0.401^*^	0.241^*^

*n.s.* not significant, *p < 0.05, **p < 0.01

## Discussion

### Patient characteristics

In this study, we reviewed the clinical parameters of 130 implants in 55 patients during ongoing long-term maintenance. We focused on the history of periodontitis and compared the parameters in two groups classified according to a history of periodontitis. A previous study on the prevalence of periodontitis revealed that moderate or severe periodontitis was observed in 64% of people over 65 years of age [19], and that the number of remaining teeth also decreased after the age of 60 [20]. Consistent with these findings, our study suggests that periodontal treatment was started at an older age in the HP group. Previous reports recommended that the follow-up period evaluating the peri-implant tissue should be 5 years or longer. The mean maintenance period of this survey was 6 years and 6 months in total. Therefore, we consider that the observation period of this study was sufficiently long-term and reasonable, resulting in a potentially predictive result [3]. The implant maintenance period of the HP group was longer than that of H group, possibly because the HP group consisted of patients with a history of good compliance who had visited the university hospital for long-term periodontal treatment and understood the importance of periodontal treatment and were likely to continue with maintenance visits after the implant treatment. However, it was expected that patients in the H group are often only partially treated and they had less experienced to receive comprehensive or long-term treatment. We hypothesized that the patients of H group might have felt less necessity of receiving maintenance because they were healthy.

A history of periodontitis has been recognized as an important risk indicator for peri-implantitis [2, 3, 21] and is known to lower the success rate during maintenance. Thus, for long-term implant stability, it is important to perform appropriate periodontal treatment [22–24].

The difference between the number of natural teeth at the first visit and at the beginning of implant treatment indicates that many teeth were extracted in the HP group compared with the H group during the active treatment period. One limitation of this study is that we did not investigate the reasons for tooth extraction. We inferred that in the HP group, many teeth were removed because of periodontitis, whereas a smaller number were extracted in the H group because of root fracture or apical periodontitis. There was no significant difference in the number of implants between the groups. However, the number of natural teeth before implant treatment was smaller in the HP group than in the H group. It is possible that the implants in these patients were used as bridge abutments for wide defects and that the size of the defect was not necessarily reflected in the number of implants.

### The severity of periodontitis and peri-implantitis

All the implants with peri-implantitis came from the HP group, not the H group, thus supporting the view that a history of periodontitis is a risk indicator for peri-implantitis. In the diagnosis of periodontitis within the HP group, it is notable that peri-implantitis increased as periodontitis become more severe. This supports the findings of a previous study that reported that the survival rate of implants is inversely proportional to the severity of past periodontitis [25]. In our study of long-term maintenance patients, the findings that the prevalence of peri-implantitis was 10.8% at the implant level and 10.9% at the patient level were similar to previous findings. It was suggested that the criteria for peri-implantitis that we adopted were reasonable. The low prevalence of peri-implantitis in this study (approximately 10%) may be related to the fact that all the subjects received maintenance therapy [26].

### Evaluation of clinical parameters

The average mPI was about 0.2 for both groups, indicating little plaque adhesion. One characteristic of patients in this study is that they understood the importance of maintenance and they received oral hygiene instruction. Although poor oral hygiene has been reported to be a risk indicator for peri-implant disease [3], our results suggest that the oral hygiene was maintained in all patients including peri-implantitis patients.

Among all the subjects, 11.5% of the total implant sites had an average PPD of more than 4 mm. Our results were comparable with previous studies, which have reported peri-implant PPDs ranging from 2.52 to 3.8 mm [27–29]. However, we believe that this study was more thorough because of its long-term follow-up. PPD in the HP group was 3.33 ± 1.07 mm, which was significantly greater than that in the H group (2.87 ± 0.48 mm). Interestingly, we found that PPD was greater in patients with a history of periodontitis than in those without this history. It has been reported that worsening periodontal disease of natural teeth affects the pocket depth of adjacent implants [30]. However, although in natural teeth there is a criterion of critical probing depth (4 mm or more) [31], there are no such reference values for implants because the site and placement depth differs and the biological width of the implant is not constant [32].

BoP has been used to evaluate inflammatory conditions of periodontal tissue [33] and can also be an important evaluation item for peri-implant tissues. To avoid diagnosing bleeding resulting from strong probing as a false positive, we set the probing pressure to 0.15 N [34]. BoP values were low, with no significant difference between the groups (H group, 0.30; HP group, 0.28). This result was similar to that of a previous study that reported a low bleeding index in the implant group with a low plaque index [35].

### Correlations between probing pocket depth around implants and age, number of teeth, extracted teeth, and maintenance period

There was no significant difference between mPI and BoP when clinical parameters were compared, and there was a significant difference for PPD only in the HP group. For this reason, we analyzed the correlation of the characteristics of patients with significant differences only for PPD. In the H group, a significant correlation with PPD was found only for the number of extracted teeth, suggesting that PPD was correlated with the number of teeth extracted due to reason that it was not periodontitis.

However, in the HP group, age, number of teeth, extracted teeth, and maintenance period all correlated with PPD. This finding suggests that the implant PPD reflects the period required for periodontitis treatment and complexity of treatment before implant placement. Although the number of teeth at the beginning of implant treatment showed only a negative correlation, the small number of teeth present indicates that a large number of teeth were extracted as a result of periodontitis, suggesting that the implant PPD is affected by this. From these results, it can be speculated that the severity of periodontitis before the implant treatment is also reflected in the PPD.

### Correlations between clinical parameters

Correlations between PPD and the two other clinical parameters, mPI and BoP, were examined in each group. Because this study targeted all patients who received maintenance, it was predicted that the hygiene around the implants in both groups would be good. However, there was a significant difference between PPD and mPI only in the HP group. It was assumed that the probing depth and oral hygiene around implant appeared to be affected each other in the HP group. In contrast with the target group of our study, there may have been a significant correlation between PPD and mPI in a patient group that had not received maintenance and that had poor oral hygiene.

A correlation between the PPD of the peri-implant tissue and lesion progression has been reported, similar to that seen with periodontitis [27]. Probing around the implant is useful for evaluating soft tissue inflammation and is considered reproducible [36]. Examination of peri-implant bleeding is reported to have higher diagnostic accuracy than probing around natural teeth [37]. However, because measurement results differ depending on the shape of the superstructure and because bleeding can occur even if peri-implant tissue is healthy, implant probing is not completely established as a method to distinguish between healthy and inflamed sites [37, 38]. As in natural teeth, we consider that peri-implant BoP should be assessed after checking for sulcus bleeding. The results of this study, especially the correlation between PPD and BoP, suggest that implant probing is useful for evaluating peri-implant tissue.

In this study, we measured clinical parameters at only one point during the maintenance period. Further investigation is needed with attention not only to the amount of parameter change over time but also to bone resorption on radiological images.

## Conclusions

We examined clinical parameters in patients receiving long-term implant maintenance. There were significant differences between the H and HP groups in age at the time of maintenance, number of natural teeth at the beginning of implant treatment, total number of extracted teeth, and maintenance. The prevalence of implant-based peri-implantitis was 10.8% of all the implants. All implants with peri-implantitis came from the HP group, and many belonged to patients with a history of severe periodontitis. The PPD around implants was significantly greater in the HP group than in the H group. We found weak positive correlations between PPD and BoP (*r*_*s*_ = 0.401, *p* < 0.05) in the H group and between PPD and BoP (*r*_*s*_ = 0.241, *p* < 0.05) and PPD and mPI (*r*_*s*_ = 0.228, *p* < 0.05) in the HP group. Our findings suggest that the clinical parameter of probing pocket depth is a critical index for the diagnosis of peri-implant disease during the maintenance period among healthy and history-of-periodontitis groups.

## Abbreviations

BoP: Bleeding on probing
mPI: Modified plaque index
PPD: Probing pocket depth

## References

[1] Esposito M, Hirsch JM, Lekholm U, Thomsen P (1998). Biological factors contributing to failures of osseointegrated oral implants. (I) Success criteria and epidemiology. Eur J Oral Sci.

[2] Ogata Y, Nakayama Y, Tatsumi J, Kubota T, Sato S, Nishida T (2017). Prevalence and risk factors for peri-implant diseases in Japanese adult dental patients. J Oral Sci.

[3] Heitz-Mayfield LJ (2008). Peri-implant diseases: diagnosis and risk indicators. J Clin Periodontol.

[4] Roos-Jansåker AM, Renvert H, Lindahi C, Renvert S (2006). Nine- to fourteen-year follow-up of implant treatment. Part III: factors associated with peri-implant lesions. J Clin Periodontol.

[5] Mombelli A, Müller N, Cionca N (2012). The epidemiology of peri-implantitis. Clin Oral Implants Res.

[6] Zitzmann NU, Berglundh T (2008). Definition and prevalence of peri-implant diseases. J Clin Periodontol.

[7] Fransson C, Lekholm U, Jemt T, Berglundh T (2005). Prevalence of subjects with progressive bone loss at implants. Clin Oral Implants Res.

[8] Koldsland OC, Scheie AA, Aass AM (2010). Prevalence of peri-implantitis related to severity of the disease with different degrees of bone loss. J Periodontol.

[9] Leonhardt A, Renvert S, Dahlén G (1999). Microbial findings at failing implants. Clin Oral Implants Res.

[10] de Waal YC, van Winkelhoff AJ, Meijer HJ, Raghoebar GM, Winkel EG. Differences in peri-implant conditions between fully and partially edentulous subjects: a systematic review. J Clin Periodontol 2013;40:266-286. 10.1111/jcpe.12013 23379540

[11] Lindhe J, Berglundh T, Ericsson I, Liljenberg B, Marinello C (1992). Experimental breakdown of peri-implant and periodontal tissues. A study in the beagle dog. Clin Oral Implants Res.

[12] Berglundh T, Zitzmann NU, Donati M (2011). Are peri-implantitis lesions different from periodontitis lesions?. J Clin Periodontol.

[13] Ladwein C, Schmelzeisen R, Nelson K, Fluegge TV, Fretwurst TI (2015). The presence of keratinized mucosa associated with periimplant tissue health? A clinical cross-sectional analysis. Int J. Implant Dent.

[14] Schwarz F, Schmucker A, Becker J (2015). Efficacy of alternative or adjunctive measures to conventional treatment of peri-implant mucositis and peri-implantitis: a systematic review and meta analysis. Int J Implant Dent.

[15] Monjie A, Aranda L, Diaz KT, Alarcón MA, Bagramian RA, Wang HL (2016). Impact of maintenance therapy for the prevention of peri-implant diseases: a systematic review and meta-analysis. J Dent Res.

[16] Armitage GC (1999). Development of a classification system for periodontal diseases and conditions. Ann Periodontol.

[17] Lang NP, Berglundh T; Working group 4 of Seventh European Workshop on Periodontology. Periimplant diseases: where are we now? Consensus of the Seventh European Workshop on Periodontology. J Clin Periodontol. 2011;38 Suppl 11:178-181. 10.1111/j.1600-051X.2010.01674.x 21323713

[18] Froum SJ, Rosen PS (2012). A proposed classification for peri-implantitis. Int J Periodontics Restorative Dent.

[19] Eke PI, Dye BA, Wei L, Thornton-Evans GO, Genco RJ; CDC Periodontal Disease Surveillance Workgroup. Prevalence of periodontitis in adults in the United States: 2009 and 2010. J Dent Res 2012;91:914-920. 10.1177/0022034512457373 22935673

[20] Müller F, Naharro M, Carlsson GE (2007). What are the prevalence and incidence of tooth loss in the adult and elderly population in Europe?. Clin Oral Implants Res.

[21] Renvert S, Giovannoli JL, Renvert S, Giovannoli JL (2012). Risk indicators. Peri-Implantitis.

[22] Hardt CR, Gröndahl K, Lekholm U, Wennström JL (2002). Outcome of implant therapy in relation to experienced loss of periodontal bone support: a retrospective 5-year study. Clin Oral Implants Res.

[23] Schwartz-Arab D, Herzberg R, Levin L (2005). Evaluation of long-term implant success. J Periodontol.

[24] Schou S, Holmstrup P, Worthington HV, Esposito M (2006). Outcome of implant therapy in patients with previous tooth loss due to periodontitis. Clin Oral Implants Res.

[25] Roccuzzo M, Bonino F, Aglietta M, Dalmasso P (2012). Ten-year results of a three-arm prospective cohort study on implants in periodontally compromised patients. Part 2: clinical results. Clin Oral Implants Res.

[26] Derks J, Tomasi C (2015). Peri-implant health and disease. A systematic review of current epidemiology. J Clin Periodontol.

[27] Lekholm U, Adell R, Lindhe J, Brånemark PI, Eriksson B, Rocker B (1986). Marginal tissue reactions at osseointegrated titanium fixtures. (II) A cross-sectional retrospective study. Int J Oral Maxillofac Surg.

[28] Karousis IK, Salvi GE, Heitz-Mayfield LJ, Brägger U, Hämmerle CH, Lang NP (2003). Long-term implant prognosis in patients with and without a history of chronic periodontitis: a 10-year prospective cohort study of the ITI Dental Implant System. Clin Oral Implants Res.

[29] Mengel R, Flores-de-Jacoby L (2005). Implants in patients treated for generalized aggressive and chronic periodontitis: a 3-year prospective longitudinal study. J Periodontol.

[30] Cho-Yan Lee J, Mattheos N, Nixon KC, Ivanovski S (2012). Residual periodontal pockets are a risk indicator for peri-implantitis patients treated for periodontitis. Clin Oral Implants Res.

[31] Lindhe J, Socransky SS, Nyman S, Haffajee A, Westfelt E (1982). “Critical probing depths” in periodontal therapy. J Clin Periodontol.

[32] Fuchigami K, Munakata M, Kitazume T, Tachikawa N, Kasugai S, Kuroda SA (2017). Diversity of peri-implant mucosal thickness by site. Clin Oral Implants Res.

[33] Greenstein G, Caton J, Polson AM (1981). Histologic characteristics associated with bleeding after probing and visual signs of inflammation. J Periodontol.

[34] Gerber JA, Tan WC, Balmer TE, Salvi GE, Lang NP (2009). Bleeding on probing and pocket probing depth in relation to probing pressure and mucosal health around oral implants. Clin Oral Implants Res.

[35] Buser D, Weber HP, Lang NP (1990). Tissue integration of non-submerged implants. 1-year results of a prospective study with 100 ITI hollow-cylinder and hollow-screw implants. Clin Oral Implants Res.

[36] Schou S, Holmstrup P, Stoltze K, Hjørting-Hansen E, Fiehn NE, Skovgaard LT (2002). Probing around implants and teeth with healthy or inflamed peri-implant mucosa/gingiva. A histologic comparison in cynomolgus monkeys (Macaca fascicularis). Clin Oral Implants Res.

[37] Luterbacher S, Mayfield L, Bragger U, Lang NP (2000). Diagnostic characteristics of clinical and microbiological tests for monitoring periodontal and peri-implant mucosal tissue conditions during supportive periodontal therapy. Clin Oral Implants Res.

[38] Jonathan HD, Klokkevold PR, Newman MG, Takei HH, Klokkevold PR, Carranza FA (2015). Supportive implant treatment. Carranza’s Clinical Periodontology.

